# Gender differences in the behavioral and subjective effects of methamphetamine in healthy humans

**DOI:** 10.1007/s00213-019-05276-2

**Published:** 2019-06-05

**Authors:** Leah M. Mayo, Elisabeth Paul, Jessica DeArcangelis, Kathryne Van Hedger, Harriet de Wit

**Affiliations:** 1 Center for Social and Affective Neuroscience, Department of Clinical and Experimental Medicine, Linköping University, Linköping, Sweden; 2 Department of Psychiatry and Behavioral Neuroscience, University of Chicago, Chicago, USA; 3 Brain and Mind Institute, University of Western Ontario, London, Canada

**Keywords:** Methamphetamine, Monetary incentive delay, Gender differences, Sex differences, Subjective effects, Psychomotor activation

## Abstract

**Rationale:**

Methamphetamine (MA) use is steadily increasing and thus constitutes a major public health concern. Women seem to be particularly vulnerable to developing MA use disorder, as they initiate use at a younger age and transition more quickly to problematic use. Initial drug responses may predict subsequent use, but little information exists on potential gender differences in the acute effects of MA prior to dependence.

**Objective:**

We examined gender differences in the acute effects of MA on subjective mood and reward-related behavior in healthy, non-dependent humans.

**Methods:**

Men (*n* = 44) and women (*n* = 29) completed 4 sessions in which they received placebo or MA under double-blind conditions twice each. During peak drug effect, participants completed the monetary incentive delay task to assess reaction times to cues signaling potential monetary losses or gains, in an effort to determine if MA would potentiate reward-motivated behavior. Cardiovascular and subjective drug effects were assessed throughout sessions.

**Results:**

Overall, participants responded more quickly to cues predicting incentivized trials, particularly large-magnitude incentives, than to cues predicting no incentive. MA produced faster reaction times in women, but not in men. MA produced typical stimulant-like subjective and cardiovascular effects in all participants, but subjective ratings of vigor and (reduced) sedation were greater in women than in men.

**Conclusions:**

Women appear to be more sensitive to the psychomotor-related behavioral and subjective effects of MA. These findings provide initial insight into gender differences in acute effects of MA that may contribute to gender differences in problematic MA use.

**Electronic supplementary material:**

The online version of this article (10.1007/s00213-019-05276-2) contains supplementary material, which is available to authorized users.

## Introduction

Methamphetamine (MA) is the second most commonly used illicit drug, behind marijuana, and MA use disorder accounts for the majority of persons entering treatment for drug use globally (DEA 2015; UNODC 2015). As such, understanding factors that contribute to MA use disorder is of high societal importance, particularly due to the host of negative consequences associated with prolonged use (Baicy and London 2007; Brecht et al. 2004). Initial investigations into the antecedents and consequences of substance use disorders, as well as many preclinical research efforts, have primarily focused on male users (for review, see Fattore et al. 2008). However, accumulating evidence suggests that, as compared with men, female stimulant drug users may be more vulnerable to developing problematic drug use patterns (Fattore et al. 2008). Specifically in regard to MA, women consume more drug than men, transition from recreational use to dependence more quickly, and are significantly younger than their male counterparts (Brecht et al. 2004; Hser et al. 2005; Rawson et al. 2005). While it is not yet evident why women are more susceptible to problematic MA use, one possible factor is their initial response to the drug, as initial responses to drugs appear to be predictive of future use across a variety of drug classes (de Wit and Phillips 2012). Therefore, understanding differences in how men and women acutely respond to MA may help to clarify why women are more vulnerable to developing MA-related problems.

Initial use of stimulant drugs such as MA is typically characterized by positive subjective effects, including increased energy, a sense of well-being, and euphoria (Cho 1990). For many drugs of abuse, including stimulants, initial positive subjective responses are associated with a greater likelihood of continued drug use (de Wit and Phillips 2012). Retrospective accounts suggest that positive responses to stimulant drugs during initial use predict a shorter latency to subsequent use (Davidson et al. 1993) and an increased risk of developing a substance use disorder (Lambert et al. 2006). Prospective accounts originating from controlled laboratory settings provide additional support. Non-dependent humans who report more pleasant, stimulant-like effects following amphetamine administration in the laboratory are more likely to take the drug again when given the opportunity in subsequent experimental sessions (Chait 1993; de Wit et al. 1986; Johanson and Uhlenhuth 1980). As such, understanding potential gender differences in the subjective effects of MA, prior to the development of dependence, may elucidate factors that contribute to differing rates of problematic use.

Variations in responses to acute doses of stimulant drugs may also be detected with objective, behavioral measures. Female rodents are more sensitive to the psychomotor effects of cocaine, amphetamine, and MA (Becker 1999; Hu and Becker 2003; Milesi-Hallé et al. 2007; Ohia-Nwoko et al. 2017; Schindler et al. 2002). While female rodents are also more sensitive to the reinforcing effects of cocaine (Lynch and Carroll 1999, 2000), this sex difference appears to be less evident with amphetamine and MA (Mattei and Carlini 1996; Schindler et al. 2002; Stöhr et al. 1998). In human laboratory studies, stimulant drugs also improve psychomotor performance (de Wit et al. 2002; Johnson et al. 2000), but there are few reports on the potential contribution of gender differences to these effects in controlled laboratory settings (White et al. 2002). However, women who use MA recreationally report taking the drug primarily to increase efficiency and productivity (Dluzen and Liu 2008). Thus, in women, sensitivity to the psychomotor-activating effects of MA combined with a desire to use the drug to enhance behavior may result in a higher likelihood of continued drug use.

In humans, acute administration of stimulant drugs increases extracellular dopamine (DA) within reward-related neurocircuitry, including the ventral striatum (Buckholtz et al. 2010; Leyton 2002). Anticipation of non-drug (e.g., monetary) reward activates these same areas (Haber and Knutson 2010; Knutson et al. 2001b). Moreover, greater striatal activation during anticipation of reward is associated with more positive subjective response to a stimulant drug in non-dependent humans (Crane et al. 2018). Thus, stimulant drugs may further potentiate responses to rewards, presumably through effects on DA neurotransmission. This effect may be especially pronounced in individuals more sensitive to the effects of the drug. Consistent with this, preclinical evidence shows that amphetamine infusion into the nucleus accumbens increases the incentive value of a non-drug reward, such that animals will work more for a sucrose reward under the influence of amphetamine (Wyvell and Berridge 2000).

In the present study, we aimed to explore how MA influences behavioral motivation in response to reward-related cues in healthy men and women, and whether those more sensitive to the behavioral effects of MA would also be more sensitive to the subjective drug effects. Participants completed four drug administration sessions in which they received methamphetamine (MA, 2 sessions) or placebo (PBO, 2 sessions) in alternating order. Subjective mood and drug responses, as well as cardiovascular drug effects, were assessed at regular intervals. During peak drug effect (30 min post-drug administration), participants completed a modified version of the monetary incentive delay (MID) task. We predicted that incentivized cues, particularly those of large magnitude, would elicit the fastest reaction times. We hypothesized that women would be more sensitive to the behavioral and subjective effects than men and that reward-related cues would act synergistically with drug effects to potentiate cue-elicited behavior.

## Materials and methods

### Study design

**Fig. 1:**
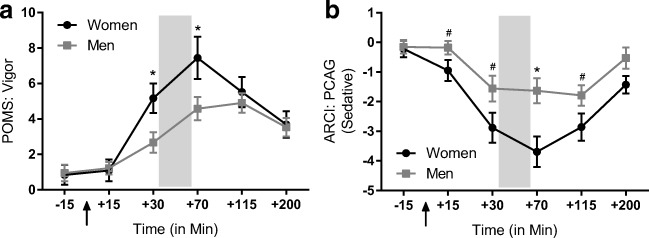
Women are more sensitive to the psychomotor-related subjective effects of methamphetamine. **a** Women report a greater increase on the Profile of Mood States “vigor” subscale following MA administration. **b** MA also produces a greater decrease on the Addiction Research Center Inventory “PCAG” scale that is sensitive to sedative-like effects. In both cases, these gender differences are evident during task administration. Lines represent baseline-corrected difference score (MA − PBO) rating for a given time point. Error bars represent standard error of the mean. Arrows indicate drug administration; MID task was completed during the time points within the shaded area; *p < 0.05, men vs women; ^#^*p* < 0.10, men vs women

Participants (*n* = 73, 29 women) completed four sessions in which they received either methamphetamine (MA, 20 mg oral) or placebo under double-blind conditions as part of a larger study investigating conditioned drug responses (Mayo and de Wit 2015). Both drug and placebo were administered twice in alternating order (MA, PBO, MA, PBO or PBO, MA, PBO, MA), and the drug order (i.e., MA first or PBO first) was counterbalanced across participants. During each 4-h-long session, participants completed a modified version of the MID task during the window of estimated peak drug effect (30 min post-administration; see Fig. 1). Cardiovascular and subjective measures regarding drug effects and mood were obtained at multiple time points through all sessions.

### Participants

Healthy men and women aged 18–35 were recruited from the community via posters, newspaper and online advertisements, and word of mouth referral. Demographic information regarding participants is reported in Table 1. Inclusion criteria were as follows: body mass index (BMI) between 19 and 26 kg/m^2^, completed high school education, fluency in English, resting blood pressure (BP) < 140/90 mmHg, current alcohol consumption < 4 standard drinks/day, and caffeinated beverage consumption < 4 units per day. Exclusion criteria were as follows: current major Axis I DSM-IV disorder (APA 2000), including current or past alcohol or drug dependence, or psychotic disorder; diagnosis of a mood disorder within the past year; ongoing treatment with psychoactive medication; cardiovascular illness; past or present medical conditions contraindicating methamphetamine use; shift work; pregnancy, nursing, or plans to become pregnant. Women not on hormonal birth control were scheduled for testing sessions only during the follicular phase of their menstrual cycle, as subjective drug effects are dampened during the luteal phase (White et al. 2002).

**Table 1:** Participant demographics and current and lifetime drug use (*N* = 73)

	***N*** (% of total)		
	Total sample	Men	Women
Gender	3 (44 M/29 W)	44 (60%)	29 (40%)
Race			
Caucasian	44 (60%)	23 (52%)	21 (72%)
African American	11 (15%)	8 (18%)	3 (10%)
Asian	11 (15%)	8 (18%)	3 (10%)
Other	7 (10%)	5 (11%)	2 (7%)
	Mean (± SD)		
Age (years)	24.8 (3.48)	25.4 (3.86)	23.7 (2.52)
Education (years)	15.7 (1.70)	15.6 (1.70)	15.8 (1.72)
BMI	22.6 (1.86)	22.9 (1.78)	22.1 (1.93)
Body weight	70.90 (9.14)	74.9 (7.98)	64.7 (7.24)
Current drug use	Mean (± SD)		
Caffeine/day	2.01 (3.66)	2.28 (4.76)	1.60 (0.66)
Cigarettes/day	3.64 (5.33)	4.07 (6.13)	2.57 (2.89)
Drinks/week	7.04 (5.30)	6.97 (5.46)	7.14 (5.16)
Marijuana/month	9.56 (13.5)	11.9 (15.0)	2.50 (1.29)
Lifetime drug use	Ever used (% of total)		
Marijuana	55 (75%)	31 (70%)	25 (86%)
Opiates	29 (40%)	15 (34%)	14 (48%)
Stimulants	23 (32%)	14 (32%)	9 (31%)
Hallucinogens	20 (27%)	14 (32%)	6 (21%)
MDMA	15 (21%)	8 (18%)	7 (24%)

Current drug use includes values only for those who regularly use; cigarettes per day for those who have used in the past month (*N* = 14; 10 M, 4 W); marijuana per month for those who have used in the past month (*N* = 16; 12 M, 4 W)

Screening included a physical examination and electrocardiogram. Participants received instructions regarding upcoming sessions and completed an abbreviated practice version of the MID task. They also read and signed the consent form which outlined the study procedures and drugs they may receive along with their effects. To prevent expectancy effects, participants were informed that they could be given placebo, a stimulant, or a sedative drug, and they were told that the purpose of the study was to investigate the effects of drugs on mood and task performance. Following completion of the study, participants were fully debriefed. The study took place at the Human Behavioral Pharmacology Laboratory at the University of Chicago Hospital and was approved by the University of Chicago Biological Science Division Institutional Review Board. Participants were paid for their participation. The present analysis was conducted with the 73 individuals (44 men, 29 women) for whom complete MID data were available from the primary study (Mayo and de Wit 2015).

### Drug

MA was administered orally. The dose used (20 mg oral; Desoxyn, Lundbeck) is safe and produces robust subjective effects (Cook et al. 1992; Martin et al. 1971; Mayo et al. 2013; Söderpalm et al. 2003). To speed up the absorption time, tablets were crushed and administered in 10 ml sugar-free syrup (Ora-Sweet, Paddock Laboratories, Minneapolis, MN) (Mayo et al. 2013; Söderpalm et al. 2003). Placebo solutions consisted of 10 ml Ora-Sweet alone and were indistinguishable from active drug by appearance or taste.

### Monetary incentive delay task

The MID task (Knutson et al. 2000, 2001a, b) was used to assess behavioral motivation (i.e., reaction time) to cues signaling potential monetary gains and losses or no monetary incentive. The task consisted of 40 trials each lasting up to 12 s. A trial consisted of a 1.5-s presentation of a cue (gain, loss, or no incentive), followed by a fixation crosshair of a variable duration (1.3–4.0 s). A white target box was then displayed for 0.5 s, signaling subjects to press the response button. After another variable interval (1.3–4.0 s), subjects were given feedback for 1.5 s about the amount of money they had won or lost during that trial, as well as their cumulative total. Trials were separated by 1.3–4.0 s. Participants were instructed to respond as quickly as possible by pressing a button every time the white target box appeared. Cues signaled potential reward (denoted by circles), loss prevention (denoted by squares), or no monetary outcome (denoted by triangles). In addition to the type of cue (win, loss, or no incentive), we also varied the magnitude of the cue. Potential reward trials included trials in which a low reward amount ($0.25; denoted by a circle with 1 horizontal line) or a high reward amount ($1.00; denoted by a circle with 3 horizontal lines) could be at stake, whereas potential loss prevention trials included trials in which a low amount ($0.25; denoted by a square with 1 horizontal line) or a high amount ($1.00; denoted by a square with 3 horizontal lines) could be lost. “Hit” trials in which the participant responded during the 0.5s target box presentation resulted in monetary gain (gain trials) or prevention of monetary loss (loss trials). If the participant responded too slow (e.g. over 0.5s), “miss” trials resulted in no gain (gain trials) or monetary loss (loss trials). Trial types were pseudorandomly ordered within each session, and the task lasted approximately 8 min. To ensure active participation during the task, participants were informed prior to the start of the task that they would receive earnings from the task in cash at the end of the session.

### Subjective and physiological measures

During each of the sessions, participants completed self-report computer-based standardized subjective mood and drug effects questionnaires at − 15, + 15, + 30, + 70, + 115, and + 200 min relative to drug administration. Measures included Profile of Mood States (McNair et al. 1971), Addiction Research Center Inventory (Haertzen 1966), and the Drug Effects Questionnaire (Johanson and Uhlenhuth 1980). We also assessed blood pressure and heart rate using portable monitors (Omron, Lake Forest, IL).

### Session procedures

Prior to the potential drug administration sessions, participants were instructed to consume their normal amount of caffeine/nicotine before sessions but to abstain from using prescription and over-the-counter drugs 24 h before and 6 h after each session, other recreational drugs 2 days before and 6 h after sessions, and marijuana 7 days before and 6 h after each session and to not operate any machinery or vehicles 6 h following each session. Compliance measures were obtained before all sessions and included a self-report questionnaire, a breath alcohol level test (Alco-Sensor III, Intoximeters, St. Louis, MO), a urine drug test (ToxCup, Branan Medical Corporation, Irvine, CA), and, for women, a pregnancy test (AimStick PBD, hCG professional, Craig Medical Distribution, Vista, CA).

Sessions were conducted from 0900 to 1300 h, with at least 48 h between sessions. After compliance tests (BAL, urine toxicology, and pregnancy), predrug measures were obtained and subjects received the drug. Study rooms were comfortably furnished with a couch, a desk and chair, a computer (for tasks and questionnaires), a television, a video player, and magazines. When participants were not completing tasks or questionnaires, they were allowed to relax, watch selected movies, or read, but were not permitted to sleep, work, or study. Cellphones and internet access were not allowed in order to minimize emotional contact outside the testing environment. The MID task was performed between time points + 30 and + 70 min after study drug administration. Participants were paid earnings from the behavioral tasks at the end of each session. They were allowed to leave once subjective and cardiovascular drug effects subsided.

### Statistics

We compared behavioral measures (reaction time, hit rate) separately for the two MA sessions and the two PBO sessions. Responses during these two sets of sessions did not differ, and so responses were averaged for the two drug sessions, as well as the two placebo sessions (Mayo and de Wit 2015). Demographic factors (age, gender, body weight, drug use history, etc.) were entered as covariates or between-subject factors in all analyses and only included in the final model when significant. We found gender to be a significant between-subject factor influencing reaction time and thus included it in subsequent analyses. We verified that the weight body mass index (BMI) was not a significant covariate (see Supplemental Results).

#### Subjective and cardiovascular effects

We created peak change scores from the six time points (relative to administration of drug, − 15, + 15, + 30, + 70, + 115, and + 200) to represent the magnitude of drug effects. Effects of MA vs PBO self-report questionnaires were assessed using the number of scales (ARCI—6, POMS—8, DEQ—5) and the treatments (MA, PBO) as within-subject factors in a RM-ANOVA with gender as a between-subject factor. We expected a main effect of scale, as each scale measures a relatively distinct subjective effect, and thus focused on scale × gender interactions. Significant interactions were followed up using Benjamini-Hochberg (B-H) post hoc tests with a false discovery rate of 0.10. Uncorrected *p* values are provided, and those that do not remain significant following B-H correction are noted. Cardiovascular effects, blood pressure, and heart rate were treated similarly. Blood pressure was represented as mean arterial pressure, calculated as follows: MAP = (systolic BP + 2 × diastolic BP) ∕ 3. In addition, although only 73 (44 men, 29 women) participants had complete behavioral data, we had complete subjective and cardiovascular data from 90 participants (50 men, 40 women). Thus, we validated our subjective and cardiovascular findings in the current 73-participant sample with the larger 90-participant sample (see Supplemental Materials).

#### MID task

Using mixed-effects analyses (also called multilevel analyses or hierarchical regression) (Blackwell et al. 2006; Hox 2010), we assessed gender differences in the influence of MA on reward processing modeled by the MID task. Mixed-effects analyses account for the nested structure of the data, where each participant contributed data in both PBO and MA sessions. They furthermore allow us to model between-subjects variation by including random effects, and assumptions of normality and homoscedasticity of residuals are relaxed.

We tested a two-level model, with drug and MID trial type nested within participants and gender as a level 2 factor. MID trial type (high gain, low gain, no incentive, low loss, high loss), drug (MA, PBO), and gender were included as fixed factors. For the MID trial type, the reference trial was defined as the no-incentive trial, comparing low and high magnitudes of gain and loss trials with those without an incentive. Interaction effects between all factors were included. Since our primary interest was to assess interaction effects between gender and drug, but not trial type, we dropped non-significant interaction effects including the trial type to make the model more parsimonious.

Significant interaction effects were followed up by estimating marginal means and their 95% confidence intervals. Furthermore, responses on the vigor subscale of the POMS and the PCAG (sedative-like) subscale of the ARCI, completed during both PBO and MA sessions, were included as fixed covariates as significant drug × gender interaction effects on these scales were found in the data at hand. However, the inclusion of the subjective data did not alter the results and was therefore excluded from further consideration. A random intercept was included to account for individual variability (see Supplementary Fig. 1 for a visual representation of variation in RT) as well as a random slope to assess variability in individual drug response. Coefficients (*β*) and their standard errors (SE) are reported, and degrees of freedom were calculated using the Satterthwaite method. All statistical analyses were carried out with IBM SPSS Statistics version 25.

### Subjective and cardiovascular measures

Subjective mood and drug effect ratings and cardiovascular effects are summarized in Table 2, in which we denote significant main effects of drug and drug × gender interactions (see also Fig. 1). Below, we focus on significant scale × gender interactions. More extensive statistical results regarding expected main effects can be found in supplemental materials.

**Table 2:** Subjective drug and mood effects at PBO and MA sessions

	Overall (*N* = 73)		Men (*N* = 44)		Women (*N* = 29)	
	PBO	MA	PBO	MA	PBO	MA
POMS						
Friendly	− *1.79* (*3.49*)	*1.40* (*4.74*)*^#^	− *1.20* (*2.92*)	*0.94* (*4.50*)	− *2.67* (*4.11*)	*2.10* (*5.09*)
Anxious	− 0.12 (1.58)	0.64 (2.86)	− 0.08 (1.64)	0.84 (2.73)	− 0.19 (1.50)	0.34 (3.07)
Elation	− **1.12** (**2.32**)	**2.35** (**3.63**)*	− 0.69 (2.45)	2.11 (3.42)	− 1.76 (1.98)	2.71 (3.96)
Anger	− 0.29 (1.90)	− 0.36 (1.41)	− 0.46 (2.04)	− 0.14 (1.40)	− 0.67 (1.62)	− 0.71 (1.37)
Fatigue	**1.01** (**2.70**)	− **0.99** (**2.39**)*	0.49 (2.76)	− 0.95 (2.27)	1.81 (2.44)	− 1.05 (2.62)
Depression	− 0.38 (1.82)	− 0.51 (2.23)	− 0.41 (2.06)	− 0.16 (1.92)	− 0.33 (1.43)	− 1.05 (2.59)
Confusion	0.40 (2.02)	− 0.14 (2.01)	0.05 (2.04)	− 0.15 (1.92)	0.93 (1.89)	− 0.14 (1.85)
Vigor	− **2.01** (**3.28**)	**2.70** (**5.25**)*^#^	− **1.43** (**3.52**)	**1.77** (**4.52**)	− **2.90** (**2.70**)	**4.10** (**6.00**)
ARCI						
A	− **0.12** (**1.33**)	**3.30** (**2.89**)*	0.02 (1.49)	3.03 (3.08)	− 0.33 (1.02)	3.71 (2.56)
MBG	− **0.18** (**1.74**)	**5.27** (**4.38**)*	− 0.07 (2.11)	4.94 (4.68)	− 0.36 (0.92)	5.78 (3.89)
LSD	**0.31** (**1.35**)	**1.05** (**1.91**)*	0.33 (1.44)	1.20 (1.92)	0.28 (1.25)	0.83 (1.89)
BG	− **1.45** (**1.64**)	**2.03** (**3.00**)*	− 1.06 (1.72)	1.98 (3.06)	− 2.03 (1.34)	2.12 (2.95)
PCAG	**2.41** (**2.83**)	− **0.08** (**3.22**)*^#^	**1.84** (**2.99**)	**0.25** (**3.41**)	**3.28** (**2.36**)	− **0.57** (**2.90**)
M	**0.90** (**1.15**)	**3.69** (**2.16**)*	0.92 (1.21)	3.63 (2.36)	0.86 (1.05)	3.79 (1.83)
DEQ						
Feel drug	**17.6** (**15.7**)	**47.2** (**23.4**)*	18.9 (17.0)	46.5 (24.5)	15.7 (13.8)	48.2 (20.3)
Like effects	**20.3** (**21.2**)	**62.0** (**22.8**)*	24.3 (23.4)	62.5 (29.2)	14.2 (15.8)	61.3 (28.5)
Dislike effects	23.3 (22.7)	25.4 (21.1)	23.9 (23.9)	24.3 (21.9)	22.6 (21.1)	27.1 (20.0)
Feel high	**10.5** (**13.7**)	**34.7** (**25.0**)*	11.6 (15.7)	32.7 (25.7)	8.74 (10.1)	37.7 (25.7)
Want more	**16.6** (**21.4**)	**58.8** (**30.7**)*	20.0 (24.1)	60.9 (31.6)	11.5 (15.5)	55.6 (29.6)
Cardiovascular						
Heart rate	− **7.87** (**9.84**)	**4.49** (**12.1**)*	− 8.44 (7.58)	3.45 (11.7)	− 7.03 (12.5)	6.02 (12.6)
Blood pressure	− **4.14** (**7.04**)	**11.2** (**10.1**)*	− 5.48 (5.44)	9.65 (11.4)	− 2.06 (8.69)	13.7 (7.11)

Values represent peak change scores from baseline at each session ± standard deviation. *POMS*, Profile of Mood States; *ARCI*, Addiction Research Center Inventory; *A*, amphetamine scale; *MGB*, morphine-Benzedrine group scale; *LSD*, lysergic acid diethylamide scale; *BG*, Benzedrine group scale; *PCAG*, pentobarbital, chlorpromazine, and alcohol group scale; *M*, marijuana scale; *DEQ*, Drug Effects Questionnaire. Blood pressure calculated as mean arterial pressure ((systolic BP + 2 × diastolic BP) ∕ 3). *p < 0.05, effect of drug; ^#^*p* < 0.05, drug × gender interaction. Bold values are significant at corrected *p* < 0.05; italicized values are significant at uncorrected *p* < 0.05

#### POMS

There was a significant drug × gender interaction for the vigor scale such that women reported a greater increase in vigor than men (*p* = 0.009; Fig. 1a). There was also a trend towards a drug × gender interaction for the friendliness scale (*p* = 0.041), again with women reporting a greater increase than men, but this did not remain significant after correction for multiple comparisons. These effects were not influenced by body weight (vigor, *p* = 0.98; friendly, *p* = 0.48) or BMI (vigor, *p* = 0.27; friendly, *p* = 0.41). Across both genders, MA produced the expected stimulant-like effects (main effect of drug (*F*(1,71) = 57.6), *p* < 0.001), including ratings of friendliness (*p* < 0.001), elation (*p* < 0.001), and vigor (*p* < 0.001), and reduced fatigue (*p* < 0.001).

#### ARCI

There was a significant drug × gender interaction on the ARCI PCAG (pentobarbital, chlorpromazine, and alcohol group; sedative-like effects) scale such that women reported a greater reduction in sedative effects than men (*p* = 0.011; Fig. 1b) following drug administration. This effect was not influenced by body weight (*p* = 0.91) or BMI (*p* = 0.55). In both genders, MA increased the following scales (main effect of drug (*F*(1,71) = 116), *p* < 0.001): A (*p* < 0.001), MBG (*p* < 0.001), BG (*p* < 0.001), M (*p* < 0.001), and LSD (*p* = 0.014; no longer significant when correcting for multiple comparisons).

#### DEQ

There was no effect of gender on any DEQ scale. MA increased DEQ ratings (main effect of drug (*F*(1,71) = 197), *p* < 0.001) of “feel drug” (*p* < 0.001), “like effects” (*p* < 0.001), “feel high” (*p* < 0.001), and “want more” (*p* < 0.001).

#### Cardiovascular effects

MA increased heart rate (*F*(1,72) = 56.4, *p* < 0.001), but there was no effect of gender and no drug × gender interaction. MA also increased blood pressure (MAP; *F*(1,71) = 140, *p* < 0.001). There was a significant effect of gender (*F*(1,71) = 5.01, *p* = 0.028) such that men had lower mean arterial pressure across both sessions, but this was unaffected by drug (drug × gender: *p* = 0.82).

### MID task

**Fig. 2:**
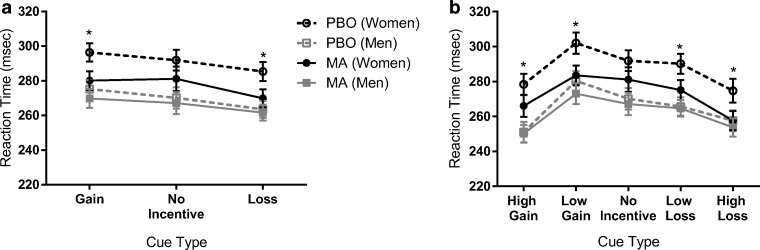
Methamphetamine speeds up reaction times in women only. **a** Across all trial types and both drug conditions, men respond faster than women. However, MA significantly reduces reaction times in women. **b** When trial types are broken down by magnitude, high-incentive trials elicit the fastest reaction times as compared with low or no-incentive trials. Again, MA reduced reaction times in women only. *N* = 73 (44 men, 29 women). PBO, placebo; MA, methamphetamine. Lines represent mean reaction times, and error bars indicate standard error of the mean

Results are presented in Tables 3 and 4 and Fig. 2. The mean reaction time across all participants, trial types, and drug conditions was 268.8 ms. The low loss trial did not significantly differ from the no-incentive trial. Participants reacted slower to the low-gain cue, but significantly faster to the high-incentive cues compared with no incentive. Men and women significantly differed in mean reaction times regardless of drug administration, with women having slower reaction times than men. MA did not have a main effect on reaction time, but significantly decreased reaction times in women only (Table 4).

**Table 3:** Estimated parameters and significance of the fixed effects modeling reaction time in the monetary incentive delay task using a mixed-effects model

	*β*	SE	*t* (df)	*p*
Intercept	*268.82*	*4.79*	*56.15* (*110*)	<*0.001*
Gender	*24.09*	*9.04*	*3.34* (*90.37*)	*0.001*
Drug	− 1.98	2.99	− 0.655 (73)	0.515
Trial type				
Low loss	− 3.49	2.34	− 1.49 (584)	0.137
High loss	− *15.48*	*2.34*	− *6.62* (*584*)	<*0.001*
Low gain	*7.78*	*2.34*	*3.33* (*584*)	*0.001*
High gain	− *16.23*	*2.34*	− *6.94* (*584*)	<*0.001*
Drug × gender	− *12.82*	*4.74*	− *2.7* (*73*)	*0.009*

Italicized values are significant at *p* < 0.05

**Table 4:** Estimated marginal means and 95% confidence intervals for the drug × gender interaction effect

	Men		Women	
	Estimated marginal mean	95% confidence interval	Estimated marginal mean	95% confidence interval
PBO	263.3	254.3–272.4	287.4	276.3–298.6
MA	261.4	252.3–270.4	272.7	261.5–283.8

*PBO*, placebo; *MA*, methamphetamine

Estimated marginal means and 95% confidence intervals around them were calculated to further understand the interaction effect between drug and gender. These results indicate that women respond significantly faster on MA compared with PBO, but there is no difference in men (Fig. 2).

## Discussion

In this study, we assessed the effect of MA on cue-elicited behavior using the MID task in healthy, non-dependent men and women. Overall, cues signaling potential monetary losses or gains, particularly those signaling high-magnitude incentives, elicited the fastest reaction times. We found that MA had a robust effect on behavior in women, producing faster reaction times across all trial types, but there was no effect of MA on behavior in men. MA produced the typical profile of subjective effects across both genders. However, we found that MA resulted in greater ratings of vigor (POMS) and reduced sedation (ARCI—PCAG scale) to a larger degree in women as compared with men. We did not find gender differences in the cardiovascular effects of MA, nor were the gender effects influenced by body weight or BMI, suggesting that the results presented are likely not attributable solely to straightforward pharmacokinetic differences. Thus, we find gender differences in the effects of MA that are consistent across behavioral and subjective measurements and as such highlight novel gender differences in the acute effects of MA that may contribute to reported gender differences in MA use and abuse.

Overall, we find that MA administration produces faster reaction times in women, but does not significantly affect behavior in men. This is in line with preclinical research suggesting that female rodents are more sensitive to the psychomotor-activating effects of MA than males (Milesi-Hallé et al. 2007; Ohia-Nwoko et al. 2017; Schindler et al. 2002). Moreover, we find that MA increased ratings of vigor (POMS) and reduced ratings of sedation (ARCI—PCAG) to a greater extent in women than in men. Our mixed-effects model did not find a significant interaction between behavior and subjective measures, suggesting that these behavioral and subjective effects may be reflective of a shared underlying construct. As a result, women show heightened sensitivity to the psychomotor-related effects of MA, regardless of whether these effects are assessed subjectively or behaviorally. This corroborates previous reports of enhanced sensitivity to MA, but not amphetamine, in healthy women (White et al. 2018).

It should be noted that we did see significant differences between genders at the placebo session, such that men responded faster to all cues in the absence of MA. Consequently, it is possible that we did not see an effect of drug in men due to a ceiling effect of them responding so quickly during the placebo session. However, the fact that high-magnitude incentives were able to elicit faster reaction times than low-magnitude incentives suggests that men were indeed capable of speedier reaction times, but that MA itself was not sufficient to reduce reaction times in these individuals. This is further supported by previous studies suggesting that monetary incentives elicit faster reaction times in men than in women (Spreckelmeyer et al. 2009), suggesting that the differences in reaction times during the placebo condition are indeed representative of a reliable gender difference in cue-elicited behavior.

We used the MID task to assess whether MA would influence behavioral responses to reward-related cues. Neuroimaging studies using the MID task have shown that anticipation of monetary gains or losses elicits activation of the ventral striatum that scales with reward magnitude (Knutson et al. 2000, 2001a, b). Moreover, multimodal brain imaging studies suggest that DA release in these areas is correlated with reward anticipatory activation (Weiland et al. 2014). Stimulant drugs also enhance striatal DA release in humans (Buckholtz et al. 2010; Oswald et al. 2007). Thus, we predicted that the dopaminergic effects of the reward cues and MA administration may act synergistically to potentiate behavioral reactions. However, we instead found that MA either increased reaction times in response to all cues (e.g., in women) or had no effect on response to any cues (e.g., in men), regardless of whether the cue signaled potential monetary loss and gain or no incentive. Thus, we failed to find a facilitation of reward-elicited behavior by MA.

It is possible that our slight modifications of the task may have influenced our findings. The more commonly used fMRI version of the MID has a titrated target presentation time to ensure that only 2/3 of trials are successfully completed (Knutson et al. 2001a). In our modified version of the task, the target presentation was fixed, and as a result, participants responded accurately at a much higher rate (approximately 98%). Thus, the reward-associated cues might have elicited greater incentivized behavior if the rewards were less frequent. However, others (Hasler et al. 2009) have assessed behavioral responses in the MID following catecholamine depletion, which reduces the amount of available dopamine, norepinephrine, and epinephrine. These authors report that catecholamine depletion in women with remitted major depression shows deficits in reward-related behavior. However, consistent with our results, catecholamine depletion produced slower reaction times across all trial types, not only incentivized trials. This study used the more standard version of the MID task, in which target presentation times are based on individual reaction times, and still found that depleting catecholamines influenced behavior across all trial types. As such, it is unlikely that our results are a consequence of these minor task modifications. Moreover, it lends further support to our findings, as modulation of dopamine in the opposite direction (i.e., a reduction via catecholamine depletion) produced slower reaction times. Interestingly, in both cases, these effects are reported only in women.

Women and men who regularly consume MA report different reasons for drug consumption. Although both genders report that availability of the drug is the primary reason for use, women report that the second most common reason is to increase energy and productivity, while men report curiosity as a reason for using (Cretzmeyer et al. 2003). Here, we find that in a fairly stimulant-naïve population (approximately 30% with previous use), women report greater increases in the psychomotor-related effects of MA. Thus, in the early stages of MA use, women may be more sensitive to MA’s ability to enhance behavioral output. Moreover, they may be more likely to consume MA again due to these psychomotor-related effects. These effects may contribute to the faster transition from initial to problematic MA use that has been reported in women (Dluzen and Liu 2008).

Mechanistic differences in the pharmacodynamics of individual stimulant drugs likely contribute to differences in their use and abuse potential. There is some evidence that MA is more effective than amphetamine in releasing serotonin and that MA is more toxic to serotonergic than to dopaminergic systems (Kuczenski et al. 1995; Sabol et al. 1995). Moreover, extensive sexual dimorphism exists in the serotonergic system between males and females (Zhang et al. 1999), suggesting that these serotonergic effects may influence males and females differently. Accordingly, women more frequently report using MA to escape emotional problems and cope with mood (Cretzmeyer et al. 2003; Semple et al. 2004) and have higher rates of comorbid depression (Hser et al. 2005). In the analysis of our larger data set (*N* = 90; 50 men, 40 women), we find that women report more positive mood-related subjective effects following MA administration, including increased ratings of friendliness, elation, and reduced depressive ratings. These effects did not reach statistical significance in the smaller sample, though the directionality of effects was in concordance. While preliminary, this may serve as initial evidence supporting the hypothesis that MA use in women may be related to the positive effect on subjective mood. Subsequent studies may provide further insight by including women with a greater range of depressive symptomatology to determine if the positive effects of MA are even more pronounced in those with greater mood deficits.

Previous studies have highlighted differences in subjective response to stimulant drugs throughout the menstrual cycle (White et al. 2002). Women in the current study who were not on hormonal birth control were only tested during the follicular phase, as subjective responses during this phase do not differ from men. This somewhat limits the interpretation of our effects to this menstrual cycle phase. Studies assessing effects of stimulant drugs on the menstrual cycle have primarily involved cocaine or amphetamine (Lukas et al. 1996; White et al. 2002). Future work may determine how similar the effects of MA are to those of amphetamine across the menstrual cycle and if behavioral effects are consistent across the menstrual cycle phase. These data may potentially highlight time periods in which women may be particularly resilient, or more vulnerable, to MA abuse or attempt at MA treatment.

A major strength of this study is the large sample size. However, this strength is somewhat limited by the fact that we only used a single dose of MA. It is not known if these effects would be apparent in higher or lower doses. In addition, we did not dose based on body weight but instead used a standard dose (20 mg), comparable with previous studies (Ballard et al. 2015; Mayo et al. 2013), though the effects reported here were not driven by differences in body weight. Moreover, genetic variation at the cytochrome P450 isozyme CYP2D6 can influence the metabolism of methamphetamine, and we did not control for this, nor do we know if this influences metabolism to different degrees in men and women (De La Torre et al. 2012). Finally, our sample included healthy young adults who may not be representative of those most vulnerable to MA use in naturalistic, real-world settings. For instance, we used oral dosing, whereas most MA users transition to smoked or intravenous administration (Brecht et al. 2004). The route of drug administration of stimulant drugs can contribute to abuse potential (Lile et al. 2011). Thus, one should take caution when extrapolating the effects reported in healthy adults to dependent MA users.

In summary, the current study highlights novel gender differences in the behavioral and subjective effects of MA in healthy, non-dependent humans. Our results suggest that women are particularly sensitive to the psychomotor-activating effects of MA during acute administration, as assessed via self-report and behavior. Heightened sensitivity to these behavioral and subjective effects in women may, in combination with other factors, promote future drug use. This may be particularly true for women who consume MA to enhance productivity and efficiency and eventually contribute to the increased MA-related negative consequences experienced by women compared with men.

## Electronic supplementary material

ESM 1 (DOCX 280 kb)
