# Supplementary material for: Gender differences in the behavioral and subjective effects of methamphetamine in healthy humans
Source: Psychopharmacology (Berl). 2019 Jun 5;236(8):2413–23. doi: 10.1007/s00213-019-05276-2 (PMC6695366; doi:10.1007/s00213-019-05276-2)
Supplement: Supplementary file 1 — (DOCX 280 kb) [file 213_2019_5276_MOESM1_ESM.docx]

**Supplementary Results**

Body weight significantly differed between men and women (*F*(1,72) = 30.8, *p* < 0.001), so this was included as a co-variate in the following analyses, though it did not significantly influence any behavioral or subjective outcome measure. As we used a standard dose (20mg), on average, men received a dose of 0.88 mg/kg and women received a dose of 0.90 mg/kg.

*MID task results (RM-ANOVA)*

To analyze behavioral data in the MID task, we used a 5 (trial type; high gain, low gain, no incentive, low loss, high loss) x 2 (drug; MA, PBO) RM-ANOVA. It revealed main effects of trial type (*F*(4,284) = 30.3, *p* < 0.001), drug (*F*(1,71) = 12.1, *p* = 0.001), and gender (*F*(1,71) = 6.53, *p* = 0.013), and a drug*gender interaction (*F*(1,71) = 7.11, *p* = 0.009). Overall, women had longer reaction times than men at the PBO session (effect of gender: *p* = 0.002) but there was no difference in reaction times on MA (effect of gender: *p* = 0.13). In women, MA reduced reaction times to high gain (*p* = 0.016), low gain (*p* = 0.001), low loss (*p* = 0.001), and high loss (*p* = 0.021) cues, but not no incentive cues (*p* = 0.060). MA did not influence responses to any cues for men (high gain, *p* = 0.086; low gain, *p* = 0.17; no incentive, *p* = 0.96; low loss, *p* = 0.96; high loss, *p* = 0.44). Body weight (*p* = 0.30) and BMI (*p* = 0.51) were not a significant co-variates.

*Subjective and Cardiovascular Measures (N = 73)*

*POMS:* MA produced the expected stimulant-like effects on the Profile of Mood States (POMS). We found the expected main effects of drug (*F*(1,71) = 57.6 *p* < 0.001) and scale (*F*(7,504) = 2.49, *p* = 0.016), as well as drug*scale (*F*(7,497 = 28.8, *p* < 0.001) and drug*scale*gender (*F*(7,497) = 4.09, *p* < 0.001) interactions. MA increased ratings of Friendliness (*p* < 0.001), Elation (*p* < 0.001), and Vigor (*p* < 0.001), and reduced Fatigue (*p* < 0.001). There was a significant drug*gender interaction for the POMS Vigor scale (*p* = 0.009) such that women reported a greater increase in vigor than men. We also found and a marginal drug*gender interaction effect on Friendliness such that women reported a greater MA-induced increase in Friendliness than men (*p* = 0.041), but this did not remain significant after correcting for multiple comparisons.

*ARCI:* There was a main effect of drug (*F*(1,71) = 116, *p* < 0.001), scale (*F*(5,355) = 22.8, *p* < 0.001), as well as drug*scale (*F*(5,355) = 60.6, *p* < 0.001) and drug*scale*gender (*F*(5,355) = 3.14, *p* = 0.009) interaction. In particular, MA increase the ARCI A (Amphetamine/stimulant-effects; *p* < 0.001), MBG (Morphine-Benzedrine Group; euphoric effects; *p* < 0.001), LSD (Lysergic Acid diethylamide; *p* = 0.014), BG (Benzedrine Group; *p* < 0.001) and M (Marijuana; *p* < 0.001) scales and reduced ratings on the PCAG (Pentobarbital, Chlorpromazine, and Alcohol Group; sedative effects; *p* < 0.001) scale. There was a significant drug*gender interaction (*p* = 0.011) such that females reported a greater reduction in sedative-like effects than men.

*DEQ:* There was a main effect of drug (*F*(1,71) = 197, *p* < 0.001), scale (*F*(4,284) = 24.8, *p* < 0.001) and a scale*drug interaction (*F*(4,284) = 41.4, *p* < 0.001). MA increased DEQ ratings of “Feel Drug” (*p* < 0.001), “Like Effects” (*p* < 0.001), “Feel High” (*p* < 0.001), and “Want More” (*p* < 0.001). There was no interaction with gender for any factor (drug*gender, *p* = 0.073; scale*gender, *p* = 0.48; scale*drug*gender, *p* = 0.98).

*Cardiovascular effects:* MA increased heart rate (*F*(1,72) = 56.4, *p* < 0.001), but there was no effect of gender (*p* = 0.33) or gender*drug interaction (*p* = 0.73). MA also increased blood pressure (calculated as mean arterial pressure; *F*(1,71) = 140, *p* < 0.001). There was no drug*gender interaction (*p* = 0.82), but there was an effect of gender (*F*(1,71) = 5.01, *p* = 0.028) such that men had lower mean arterial pressure across both sessions.

*Subjective Effects (N = 90)*

We verified that these effects remained when including the full study sample (n = 90), to ensure that the gender differences were not a consequence of removing subjects without complete behavioral data.

*POMS*: We found main effects of drug (*F*(1,88) = 84.6, *p* < 0.001) and scale (*F*(7, 616) = 2.48, *p* = 0.016), as well as significant interactions: drug*gender (*F*(1,88) = 4.30, *p* = 0.041; drug*scale (*F*(7,616) = 43.6, *p* < 0.001); drug*scale*gender (*F*(7,616) = 7.95, *p* < 0.001). As before, MA increased Friendliness (*p* < 0.001), Elation (*p* < 0.001), and Vigor (*p* < 0.001) and reduced Confusion (*p* < 0.01) and Fatigue (*p* < 0.001). With this larger sample size, we also detected significant gender*drug interactions such that women reported more Vigor (*p* = 0.001), Friendliness (*p* = 0.010), and Elation (*p* = 0.013), and less Fatigue (*p* = 0.030), Confusion (*p* = 0.030), and Depression (*p* = 0.032) as compared to men.

*ARCI:* We found main effects of drug (*F*(1, 88) = 130, *p* < 0.001) and scale (*F*(5,440) = 24.7), *p* < 0.001) and interactions between drug*scale (*F*(5,440) = 74.4, *p* < 0.001) and drug*scale*gender (*F*(5,440) = 5.71, p < 0.001), but not drug*gender (*p* = 0.62) or scale*gender (*p* = 0.78). MA influenced ratings on the A (*p* < 0.001), MBG (*p* < 0.001), LSD (*p* = 0.029), BG (*p* < 0.001), PCAG (*p* < 0.001), and M (*p* < 0.001) scales. There was a drug*gender interaction for PCAG (*p* = 0.001), ARCI-A (*p* = 0.041), and BG (*p* = 0.048) scales, such that women reported greater ratings on the A and BG scales, and less on the PCAG (sedative-like) scale.

*DEQ:* There were main effects of drug (*F*(1,88) = 215, *p* < 0.001) and scale (*F*(4,352) = 33.8, *p* < 0.001) and a drug*scale interaction (*F*(4,352) = 50.8, *p* < 0.001). MA increased ratings of “Feel Drug” (*p* < 0.001), “Like Effects” (*p* < 0.001), “Feel High” (*p* < 0.001), and “Want More” (*p* < 0.001). There were no other interaction effects (drug*gender, *p* = 0.13; scale*gender, *p* = 0.21; drug*scale*gender, *p* = 0.92).

*Cardiovascular effects.* MA significantly increased heart rate (*F*(1,88) = 71.5, *p* < 0.001) but there was no effect of gender (*p* = 0.11) or gender*drug interaction (*p* = 0.73). MA also increased mean arterial blood pressure (*F*(1,89) = 58.0, *p* < 0.001). There was a main effect of gender (*p* = 0.011) such that men had lower MAP overall, but this was not affected by drug (drug*gender interaction: *p* = 0.29).

|  | **Overall** (N = 90) | |  | **Males** (N = 50) | |  | **Females** (N = 40) | |
| --- | --- | --- | --- | --- | --- | --- | --- | --- |
|  | *PBO* | *MA* |  | *PBO* | *MA* |  | *PBO* | *MA* |
| **POMS** | | | | | | | | |
| *Friendly* | **-1.88 (3.57)** | **1.65 (4.71)*#** |  | **-1.29 (2.89)** | **0.93 (4.42)** |  | **-2.61 (4.20)** | **2.55 (4.95)** |
| *Anxious* | -0.16 (1.69) | 0.53 (2.81) |  | -0.05 (1.82) | 0.88 (2.65) |  | -0.30 (1.52) | 0.10 (2.96) |
| *Elation* | **-1.23 (2.68)** | **2.64 (3.50)*#** |  | **-0.81 (2.48)** | **2.09 (3.28)** |  | **-1.76 (2.86)** | **3.34 (3.67)** |
| *Anger* | -0.22 (1.77) | -0.32 (1.37) |  | -0.01 (1.92) | -0.12 (1.34) |  | -0.48 (1.55) | -0.56 (1.38) |
| *Fatigue* | **1.16 (2.90)** | **-1.01 (2.83)*#** |  | **0.54 (2.68)** | **-0.67 (2.58)** |  | **1.94 (3.00)** | **-1.43 (3.09)** |
| *Depression* | **-0.28 (1.82)** | **-0.52 (2.07)#** |  | **-0.38 (2.08)** | **-0.15 (1.86)** |  | **-0.15 (1.44)** | **-0.99 (2.24)** |
| *Confusion* | **0.54 (2.09)** | **-0.21 (1.84)*#** |  | **0.16 (1.97)** | **-0.06 (1.73)** |  | **1.01 (2.17)** | **-0.41 (1.99)** |
| *Vigor* | **-2.25 (3.42)** | **2.81 (5.34)*#** |  | **-1.52 (3.34)** | **1.60 (4.479** |  | **-3.16 (3.34)** | **4.33 (5.98)** |
| **ARCI** | | | | | | | | |
| *A* | **-0.08 (1.35)** | **3.34 (2.81)*#** |  | **0.06 (1.45)** | **2.95 (3.03)** |  | **-0.27 (1.21)** | **3.85 (2.45)** |
| *MBG* | **-0.20 (1.89)** | **5.24 (4.45)*** |  | -0.11 (2.06) | 4.78 (4.69) |  | -0.31 (1.68) | 5.82 (4.10) |
| *LSD* | 0.33 (1.37) | 0.93 (1.95) |  | 0.39 (1.47) | 1.10 (2.00) |  | 0.26 (1.23) | 0.73 (1.89) |
| *BG* | **-1.47 (1.75)** | **2.13 (2.95)*#** |  | **-1.10 (1.71)** | **1.81 (3.03)** |  | **-1.95 (1.72)** | **2.53 (2.84)** |
| *PCAG* | **2.57 (3.00)** | **-0.15 (3.18)*#** |  | **1.83 (2.90)** | **0.35 (3.32)** |  | **3.51 (2.90)** | **-0.78 (2.92)** |
| *M* | **0.98 (1.17)** | **3.58 (2.12) *** |  | 2.95 (3.03) | 3.51 (2.36) |  | 1.00 (1.12) | 3.66 (1.80) |
| **DEQ** | | | | | | | | |
| *Feel Drug* | **17.7 (16.5)** | **46.1 (23.4)*** |  | 18.4 (16.4) | 45.3 (25.39 |  | 16.8 (16.8) | 47.2 (20.9) |
| *Like Effects* | **20.5 (20.9)** | **61.6 (29.3)*** |  | 24.0 (22.8) | 61.7 (29.6) |  | 16.0 (17.5) | 61.6 (29.2) |
| *Dislike Effects* | 23.2 (24.4) | 24.8 (21.2) |  | 22.9 (24.1) | 23.0 (21.8) |  | 23.6 (25.1) | 27.1 (20.5) |
| *Feel High* | **10.9 (14.7)** | **33.7 (24.3)*** |  | 11.2 (15.1) | 31.4 (25.4) |  | 10.5 (14.3) | 36.6 (22.9) |
| *Want More* | **17.0 (20.7)** | **58.4 (30.6)*** |  | 20.9 (23.4) | 58.7 (32.2) |  | 12.2 (15.9) | 58.0 (30.6) |
| **Cardiovascular Effects** | | | | | | | | |
| *Heart Rate* | **-7.36 ( 9.68)** | **4.6 (12.2)*** |  | -8.48 (7.58) | 3.12 (11.7) |  | -5.96 (11.7) | 6.61 (12.6) |
| *Blood Pressure* | **-4.09 (6.69)** | **13.4 (21.1)*** |  | -5.74 (5.29) | 9.50 (11.0) |  | -2.15 (7.66) | 18.1 (28.2) |
| ***Supplemental Table 2:*** *Values represent peak change scores at each session ± SD. *significant effect of drug; #significant gender*drug interaction. Blood pressure was calculated as mean arterial pressure: MAP = (systolic BP + 2 x diastolic BP)/3* | | | | | | | | |


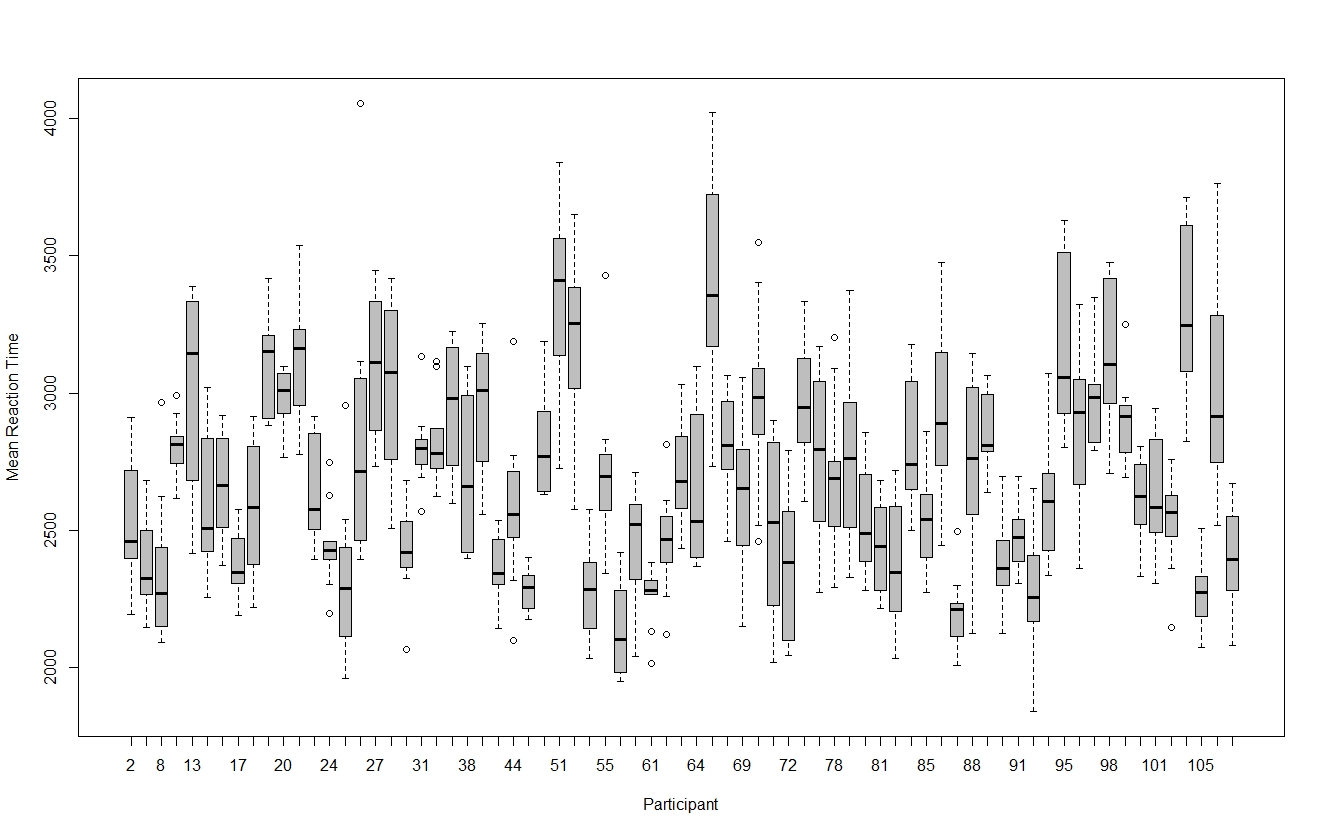


Supplementary Figure 1. Variation between participants in mean reaction time across drug and trial type.
